# Pooled prevalence of depressive symptoms among medical students: an individual participant data meta-analysis

**DOI:** 10.1186/s12888-023-04745-5

**Published:** 2023-04-14

**Authors:** Depression in Medical Students Research Group, João Pedro Gonçalves Pacheco, Adomas Bunevicius, Afiong Oku, Alan Shindel, Albina Rodrigues Torres, Ana Margareth Siqueira Bassols, Ana Teresa de Abreu Ramos-Cerqueira, Antonio Fernando, Arune Katkute, Atilla Senih Mayda, Balakrishnan Nair, Benjamin N. Breyer, Bilal Bakir, Brian Kelly, Chaisiri Angkurawaranon, Chandrashekhar Sreeramareddy, Chinthaka Samaranayake, Coumaravelou Saravanan, Cristina Marta Del-Ben, Darko Hinic, Deborah Goebert, Dragana Ristic-Ignjatovic, Eiad Al-faris, Elaine Chang, Epari Venkatarao, Ewa Helena Mojs, Farid Fayez Youssef, Gan Huang, Inesa Buneviciene, Jessica Ashley Gold, Jim Smith, Juan Enrique Berner, Katarzyna Warchol-Biedermann, Kirsten Matthews Wilkins, Maciej Walkiewicz, Maria Cristina Pereira Lima, Megan Wolf, Miles Bore, Muhamad Saiful Bahri Yusoff, Nazan Karaoglu, Omar Mousa, Patricia Lacerda Bellodi, Robert Rohrbaugh, Ruchi Singh, Sandhya Gupta, Sergio Baldassin, Sherina Mohd-Sidik, Tal Peleg-Sagy, Tan Siew Tin, Thelma Quince, Wafaa Yousif Abdel Wahed, Xinran Hu, Zhening Liu, Wilson Baldin Zatt, Kenneth Lo, Wilson Tam

**Affiliations:** 1 Department of Neuropsychiatry, Federal University of Santa Maria, Santa Maria, Brazil; 2 Department of Food Science and Nutrition, The Hong Kong Polytechnic University, 11 Yuk Choi Road, Hung Hom, Kowloon, Hong Kong SAR, China; 3 Research Institute for Smart Ageing, The Hong Kong Polytechnic University, 11 Yuk Choi Road, Hung Hom, Kowloon, Hong Kong SAR, China; 4 Alice Lee Centre for Nursing Studies, Yong Loo Lin School of Medicine, National University of Singapore, Singapore, Singapore

**Keywords:** Medical students, Depression, Individual data, Meta-analysis

## Abstract

**Background:**

The methodological choice of aggregated estimates for meta-analysis may be notable for some common drawbacks, including variations in the cut-off values of depression, and lower statistical power for analyzing the associated factors. The study aimed to refine the precision of previous findings on the prevalence of depressive symptoms among medical students, through gathering individual participant data (IPD) as identified from our previous reviews.

**Material and methods:**

In the present study, we searched MEDLINE, EMBASE, PsycINFO, WanFang, Scielo and LILACS to identify published systematic reviews and meta-analyses up to March 2018, then individual data was requested for further analysis (PROSPERO registration: CRD42018091917). The participants’ age, sex, year of study, scores for depressive symptoms, and other predictor variables were requested. To pool the prevalence from the included studies, random-effects model (two-step method) was used. Multiple linear regression was used to examine the associated factors on the depression z-scores (one-step method).

**Results:**

Of the 249 studies, the datasets of 34 studies were included. The crude prevalence was 19.4% (95% CI: 18.8%, 19.9%) by one-step method and the pooled prevalence was 18.1% (95% CI: 14.1%, 22.1%) by two-step method. Multiple linear regression revealed that being a female, older age, and senior year of study were significantly associated with the z-score.

**Conclusion:**

The pooled prevalence of depressive symptoms from the Individual Participant Data (IPD) meta-analysis was lower than the previous meta-analyses using aggregated data. Age, sex, and year of study were significantly associated with the depression z-score. IPD meta-analysis may provide a more accurate estimation of disease burden, and allow verification of associated factors.

**Supplementary Information:**

The online version contains supplementary material available at 10.1186/s12888-023-04745-5.

## Introduction

Depression is a common mental disorder affecting more than 264 million people globally [1]. Common symptoms of a depressive episode include depressed mood, loss of interest or pleasure in activities, changes in appetite, sleeping problems, loss of energy, feeling worthless or guilty, difficulty in thinking, and thoughts of death [2]. People suffering from depression may function poorly at work, in school, and within their family [1]. Additionally, depression is an important risk factor for suicide [3].

Studying at university is a transitory period in life from adolescence to adulthood, during which students have to make important life decisions. Students often experience pressure from economic stress, academic demands, and interpersonal relationships [4]. Among undergraduate programs, medicine is one of the most stressful, given the heavy workloads and high expectations from society [5]. Medical students have been reported to exhibit heightened psychological morbidity, daytime somnolence, impaired social interactions, and compromised general wellbeing, leading to mental health problems such as anxiety, stress, depression, suicidal ideation, and burnout [6], and these problems may appear as early as the first year of medical school education [7, 8].

The prevalence of depressive symptoms among medical students has been estimated from primary studies to vary from 1.4% to 80.6% [9, 10]. The pooled prevalence of such symptoms has been reported in systematic reviews and meta-analyses [5, 11, 12], some of which have explored factors including age, sex, geographical region, and sample size [5, 11]. Despite their enlightening insights from previous studies, the methodological choice of aggregated estimates from primary studies to compute the pooled estimate may be notable for some common drawbacks. Firstly, variations in the cut-off values of depression, e.g. using > 4 (mild depression) or > 9 (moderate depression) as cut-off for Patient Health Questionnaire-9 (PHQ-9) [13], might lead to inaccurate reporting of prevalence, i.e. a lower cut-off score would lead to a higher prevalence, and that may misestimate the disease burden of depression. Secondly, the use of aggregated data for analyzing the associated factors (through subgroup analysis or meta-regression) might lower its statistical power.

To address the research gap, our meta-analysis aimed to refine the precision of previous findings on the prevalence of depressive symptoms among medical students, through gathering individual participant data (IPD) as identified from our previous reviews [5, 14]. In addition, we can verify the associated factors of depressive symptoms among medical students with a larger sample size of individual data, which is another added value of the present study.

## Methods

An IPD meta-analysis has been registered in the PROSPERO register of systematic reviews (CRD42018091917). This paper was written in accordance with the PRISMA statement for IPD [15].

### Eligibility criteria

Original studies reporting the prevalence of depressive symptoms and its associated factors were included in the current study. Studies were excluded if (a) depression or depressive symptoms among medical students were not examined; (b) full texts were unavailable; or (c) no IPD were provided by their authors; or (d) they were found to have used self-developed or non-validated metrics, and if they contained no email addresses of the corresponding authors.

### Search strategy

Primary studies were identified from among our published meta-analysis (MA) and one overview of systematic reviews (SRs) on the topic previously with records up to 13 March 2018 [5, 14], and the primary studies were essentially identified from these two studies. A search strategy was developed for MEDLINE, EMBASE, PsycINFO, WanFang, Scielo and LILACS from our previous studies. For details of the strategy, reference may be made to previous publications [5, 14].

### Study selection

Upon identification, the full texts of the primary studies were examined to determine whether depressive symptoms had been measured through validated tools such as the Beck Depression Inventory (BDI) and Centre for Epidemiologic Studies Depression Scale (CES-D). If the depressive symptoms were measured through validated tools and email addresses were available for the corresponding authors, the studies would be included for requesting the data through the corresponding authors; otherwise, the studies would be excluded.

### Data collection

For eligible studies with their corresponding authors’ email addresses, communication was initiated to request anonymous IPD on a Microsoft Excel template via email (sent in March and April 2018). Data were requested about the participants’ age, sex, year of study, scores for depressive symptoms, and other predictor variables. A two-month wait period was allowed for the authors’ response; by the end of the first four weeks, non-responsive authors were contacted for a second time. The IPD obtained across studies were combined into a consistent format with Microsoft Excel. A predefined data-cleaning procedure was adopted, in which selected variables present in at least 40% of the datasets were subjected to further analysis.

### Risk of bias assessment

The risk of bias for the included studies was assessed with the tool developed by Hoy and colleagues [16]. To assess internal and external validity, the tool included 10 items, each of which presented ratings of the risk as ‘low’ (scored as 1) or ‘high’ (scored as 0). The overall risk of bias for each study was determined by the total score.

### Data analyses

For IPD analysis, there are two common approaches, namely the two-step or one-step method. For two-step method, data cleaning was followed by the adoption of a standard or commonly used cut-off value for each scale (Table 1). The depressive status of each student was dichotomized as ‘yes’ or ‘no’ and then the prevalence was computed for the study. To pool the prevalence from the included studies, a two-step method was used [17]. Firstly, the total number of students and events were extracted. Secondly, a random-effects model was used to combine the prevalence through Review Manager 5.4. Pre-defined subgroup analyses were conducted for sex, year of study, study region, and assessment tools.

**Table 1:** Cut-off value for depressive cases among the scales

Measurement tool	Cut-off score adopted in this meta-analysis^a^
Beck Depression Inventory (BDI)—I/II	≥ 20
Centre for Epidemiologic Studies Depression Scale (CES-D)	≥ 16
Depression Anxiety Stress Scales (DASS) ‐ 21/42	≥ 14
General Health Questionnaire (GHQ)‐12	≥ 9
Hospital Anxiety and Depression Scale (HADS)	≥ 11
Kutcher Adolescent Depression Scale (KADS)	≥ 9
Patient Health Questionnaire (PHQ)‐2	≥ 3
Patient Health Questionnaire (PHQ)‐9	≥ 10

BDI [18], CES-D [19], DASS-21 [20], DASS-42 [21], GHQ-12 [22], HADS [23], KADS [24], PHQ-2 [25], PHQ-9 [13]

^a^Reference for the cut-off score

For one-step method, it was adopted to examine the data from different studies as a single consolidated large dataset [17]. The raw depression scores from each study were converted into z-scores through subtraction by the average value of the study sample and then division by the standard deviation. Multiple linear regression was used to examine the effect of sex, age, year of study, study region, and measurement tool on the z-scores, backward selection method was used to determine the final model. All analyses were performed with IBM SPSS Statistics 26.

Although it was shown that the one-step and two-step approach would often give similar results [26], the one-stage method has become more popular over the past decade as it allows all studies to be analyzed simultaneously and avoids the assumption of normally distributed study effect estimates with known variances that is usually made in the second stage of the two-stage approach [27]. A *p*-value < 0.05 was considered as statistically significance.

## Results

Of the 249 studies [5], 197 provided email addresses, based on which contact was attempted and 75 responses were received. Nine authors declined to provide the data or lost contact after the initial reply, 14 authors implied the unavailability of the datasets, and one redirected us to his collaborators, but no response was received from them. Among those providing positive responses, 14 were unable to provide the data because they lost the labelling or coding of the data or because of other reasons. Finally, the datasets of 34 studies (from 37 publications) were included in this meta-analysis (Supplementary Fig. 1).

### Characteristics of the included studies

The 34 included studies (3 from Africa, 9 from Asia, 8 from Europe, 2 from Oceania, 7 from South America, and 5 from the United States) are outlined in Table 2. A total of 18,030 students from 34 studies were included. Examination of the tools for measuring depressive symptoms reveals the following: 10 for BDI, 3 for CES-D scale, 6 for Depression Anxiety Stress Scales (DASS) -21 or -42, 3 for General Health Questionnaire (GHQ)-12, 3 for Hospital Anxiety and Depression Scale (HADS), 1 for Kutcher Adolescent Depression Scale (KADS-6), and 8 for Patient Health questionnaire (PHQ)-2 or -9. Based on standard cut-off values in Table 1 for each scale, the crude prevalence was 19.4% (95% CI: 18.8%, 19.9%). The risk of bias of the included studies were in general good with a median score of 8, i.e. with 8 out of 10 items rated as low risk.

**Table 2:** Characteristics and study quality of primary studies included for individual participant data meta-analyses

Authors	Country	Tool	Sample size	Prevalence (Standard Cut-off)	Risk of Bias^a^
Abdel Wahed 2016	Egypt	DASS21	442	45.2%	8
Al-Faris 2012	Saudi Arabia	BDI	435	23.4%	9
Angkurawaranon 2016	Thailand	PHQ9	1,014	6.5%	7
Baldassin 2008	Brazil	BDI	480	9.4%	8
Bassols 2014	Brazil	BDI	232	3.9%	7
Berner 2014	Chile	GHQ-12	384	6.5%	7
Bore 2016	Australia	DASS21	127	26.8%	8
Bunevicius 2008	Lithuania	HADS	331	14.2%	8
Del-Ben 2013	Brazil	BDI	150	2.0%	8
Goebert 2009	US	CESD	1,607	20.7%	10
Gold 2015 + Data	US + Others	PHQ-2	473	17.7%	7
Güleç 2005	Turkey	BDI	684	22.8%	8
Iqbal 2015	India	DASS42	353	36.5%	8
Karaoglu 2011	Turkey	HADS	485	28.0%	8
Leao 2011	Brazil	BDI	155	3.2%	8
Manaf 2016	Malaysia	PHQ9	206	27.2%	7
Mayda 2010	Turkey	BDI	213	32.4%	8
Mojs 2015	Poland	KADS6	414	6.7%	8
Mousa 2016	US	PHQ2	336	16.4%	7
Oku 2015	Nigeria	GHQ-12	450	2.0%	9
Peleg-Sagy 2012	Israel	CESD	201	48.2%	8
Quince 2012	UK	HADS	2,446	6.5%	9
Ristić-Ignjatović 2013	Serbia	BDI	699	5.8%	7
Samaranayake 2011	New Zealand	PHQ9	575	11.5%	8
Saravanan 2014	Malaysia	DASS21	358	14.8%	8
Sherina 2005 + Tin 2015	Malaysia	PHQ9	537	16.8%	8
Shindel 2011 + Smith 2010	US	CESD	2,306	41.2%	8
Shriyan 2011	India	DASS42	50	12.0%	8
Sreeramareddy 2007	Nepal	GHQ12	402	59.0%	9
Torres 2016	Brazil	BDI	479	7.9%	6
Wolf 2017	US	PHQ-2	331	8.2%	7
Youssef 2016	Trinidad & Tobago	PHQ9	389	37.3%	8
Yusoff 2011	Malaysia	BDI	92	2.2%	8
Yusoff 2013	Malaysia	DASS21	194	29.9%	8
		Total	18,030	19.4%	Median = 8

Remarks: *BDI* Beck Depression Inventory, *CES-D* Center for Epidemiological Studies-Depression, *DASS-21/42* Depression Anxiety Stress Scales-21 items/ 42 items, *GHQ-12* General Health Questionnaire-12 items, *HADS* Hospital anxiety and depression scale, *KADS* Kutcher Adolescent Depression Scale, *PHQ-2/-9* Patient Health questionnaire-2 items / 9 items

^a^It is the total number of items rated as “Low” risk (scored as 1)

### Results from the two-step method

**Fig. 1:**
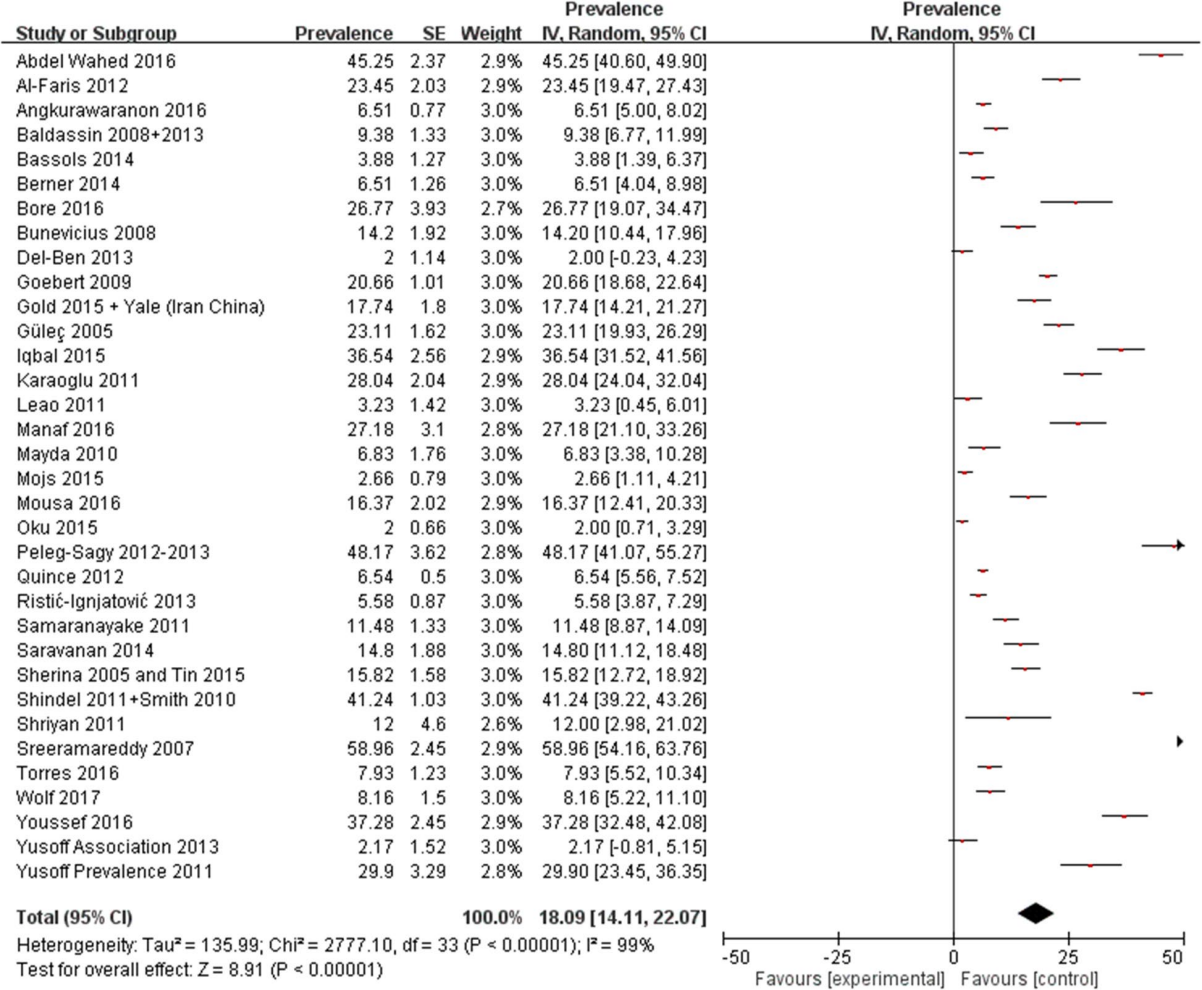
Pooled prevalence of depressive symptoms computed from the reported prevalence from each included study as derived from the two-step method

As an overview, the pooled prevalence of depressive symptoms among medical students from the included studies as derived from the two-step method was 18.1% (95% CI: 14.1%, 22.1%) (Fig. 1). This was lower than the pooled prevalence computed from the reported prevalence from each paper, i.e., 27.6% (95% CI: 22.2% to 33.0%) and there was no overlapping of their 95% confidence intervals (Supplementary Fig. 2).

In terms of sexes, the values of pooled prevalence for males and for females were 16.1% (12.4%, 19.8%) and 18.5% (14.0%, 22.9%), between which no subgroup difference was observed (*p* = 0.42) (Supplementary Fig. 3). In terms of the years of study, the pooled prevalence for the first-, second-, third-, fourth-, fifth-, and sixth-year students were respectively 19.6% (14.4%, 24.7%), 19.6% (13.0%, 26.2%), 20.6% (14.2%, 26.9%), 20.2% (14.6%, 25.7%), 14.8% (10.4%, 19.2%), and 6.9% (4.3%, 9.5%). Significant differences were observed between the subgroups (*p* < 0.001); it was observed that the prevalence of the sixth-year students was lower than their juniors and its 95% CI was also lower than all the 95% CIs of other year (Supplementary Fig. 4).

In terms of study regions, the pooled prevalence for Africa, Asia, Europe, Oceania, North America, and South America were respectively 23.6% (0%, 66.0%), 25.0% (15.3%, 34.6%), 12.2% (7.2%, 17.2%), 18.7% (3.7%, 33.6%), 23.6% (12.5%, 34.7%), and 5.5% (3.3%, 7.8%). A noteworthy finding is that the prevalence in South America was lower than other regions and its 95% CI was also lower than all the 95% CIs of other regions (Supplementary Fig. 5). In terms of assessment tools, the pooled prevalence for the BDI, CES-D scale, DASS, GHQ, HADS, KADS, and PHQ were respectively 11.8% (7.0%, 16.6%), 31.0% (10.8%, 51.5%), 27.7% (16.4%, 39.1%), 22.3% (0.0%, 45.1%), 16.2% (3.6%, 28.7%), 2.9% (1.3%, 4.5%), and 13.7% (9.2%, 18.3%). Significant differences (*p* < 0.001) were observed between the subgroups (Supplementary Fig. 6).

### Results from the one-step method

Following conversion of raw depression scores into z-scores, multiple linear regression revealed that being a female (β = 0.098, *p* < 0.001), one year older (β = 0.006, *p* = 0.037), and one year of study higher (β = –0.031, *p* < 0.001) were significantly associated with the z-score, whereas the study region and measurement tool were not (Table 3), and they were removed from the model.

**Table 3:** Association of age, sex and year of study to depression z-score

**Variable**	**Regression Coefficient (95% CI)**^a^	***p*-value**
Sex (Male as reference)	0.098 (0.065, 0.132)	< 0.001
Age	0.006 (0.000, 0.011)	0.037
Year of Study	-0.031 (-0.042, -0.020)	< 0.001

^a^Backward selection method was used and the regression coefficients for Region and Measurement Tool were not statistically significant, so they were removed from the model

## Discussion

An IPD meta-analysis was conducted to examine the prevalence and associated factors of depressive symptoms among medical students. The pooled prevalence using IPD among medical students was found from our meta-analysis to be 18.1% (95% CI: 14.1%, 22.1%). Based on the two-step method (using standard cut-off values for depression), significant differences were observed for year of study, study region, and measurement tool, but not for sex. However, based on the one-step method (converting continuous assessment scores of depressive symptoms into z-scores), multiple linear regression suggested that age, sex, and year of study were significantly associated with the depression z-score, but the study region and measurement tool were not. It is remarkable that since the cut-off values of depression varied across studies, the estimation of prevalence by simply using aggregate data might not be accurate. The strength of an IPD meta-analysis is illustrated by the standard cut-off value to classify depression in this paper.

The estimated prevalence (18.1%) using the one-step method is lower than not only the pooled prevalence (27.6%) from our included studies using the 2-step method, but also the pooled prevalence from the previous meta-analyses, i.e., 27.0% [5], 27.2% [11], 28.0% [12], and 30.6% [28]. Although our analysis was conducted based on data from only around 15% (37/249) of the total number of papers were acquired, the pooled prevalence based on 2-step method did not differ much with the previously published meta-analysis. Therefore, the representativeness of studies might not be the major cause of the difference between the estimates from the 1-step and 2-step method, one potential explanation of the difference is that the former considered each subject had the same weighting but the latter still assigned the weighting based on the inverse of the variance from the estimate in each study.

Our results have shown that female medical students exhibited significantly higher depression z-scores than their male counterparts. This finding has been echoed in the literature [14], alongside insights into sex disparities in medical education [29]. Dahlin et al. reported that female medical students were subjected to sex discrimination [30], for which they would therefore seek psychological help more often than their male counterparts [31]. Besides, a survey conducted in four medical schools United States has found that being female was the most significant risk factor for experiencing sexual harassment among medical students [32], which might in turn associate with higher psychological distress [33]. In a qualitative study [34], female students felt that they struggled to define their roles in the wards and experienced different workplace relationships compared with male students; thus, experiencing higher level of depression. From a biological perspective, sex difference has been found depression-related gene expression, neuroplasticity, and immune signatures and are in opposite directions for some of the parameters (e.g. synapse-related genes increased in women with depression and were decreased in men), which may also account for the sex-specific prevalence of depression [35].

In terms of age, older medical students have been found to exhibit significantly higher depression z-scores after adjusting the effect from sex and year of study. This finding lends corroborative evidence to the literature. Rotenstein et al. have reported a 0.2% increase in the prevalence of depression for every year of increase in age [11]. Dyrbye et al. have found that fourth-year medical students aged above 24 would exhibit significantly higher odds (OR = 1.33) of being in a depressive status than their juniors [36]. Chen et al. posited that higher prevalence originated from more stressful events confronted by older students, such as employment, finances, graduation, and marriage-related pressures [37].

Our results have also revealed that medical students in earlier years of study would exhibit higher depression z-scores. Similar findings have been reported by Quince et al., who found higher mean depression scores among preclinical (i.e. first- to third-year) medical students than their clinical (i.e. fourth- to sixth-year) counterparts [38]. Saravanan and Wilks also posited that the transition from the pre-university to the university might underlie the higher level of depression among first-year medical students [39, 40]. Aktekin and Akdemir also found that the mental health of the students was adversely affected in the first year of medical school education [7, 8]. While our meta-analysis has not found differences between regions, previous studies have suggested that Asian medical students deal with greater stress, possibly because of the cultural emphasis on diligence and filial piety as inculcated by Confucian values and concomitant high expectations of their parents, teachers, and themselves [41]. Besides, we have noted the controversy that both "older medical students" but "medical students in earlier years of study" exhibit higher depression. We suspect that the controversy is due to the different system of medical schools. For example, students can enroll to medical school directly after A-level examination in United Kingdom system, so probably one can enroll at the age of 18–19. However, students in United States are required to have a first degree before they can enroll to medical school, therefore, their age could be 21–22 at least. Taken together, first year medical students may have experienced more depressive symptoms if they entered medical school at an older age. This may explain the finding from multiple linear regression, i.e. age and year of study were both significant predictors of the depression z-score.

The strengths of our IPD meta-analysis are threefold: its large sample size with data from more than 18,000 medical students; the adoption of commonly used cut-off values for the scales characterizing depressive cases; and the use of a consistent method for analyzing data from different studies. However, some limitations are noteworthy. Firstly, the generalizability of data was limited by only acquiring individual level data from 15% (37/249) of the total number of papers and only involving 19 countries worldwide, it might introduce bias due to the non-responses. Secondly, different scales were used in the included studies, across which possible variances in definitions might still be present and they might use different cut-off due to cultural difference, thereby using a standard cut-off might increase the false negative rates. Thirdly, as self-reported instruments were used, recall bias and reporting bias were inevitable. Fourthly, the analysis included only common factors (i.e., age, sex, year of study, and region), thus potentially meaning the omission of some factors. Lastly, some studies lacked data on specific groups: studies from Africa and North America had no data on sixth-year students.

To conclude, the pooled prevalence of depressive symptoms from the IPD meta-analysis was 18.1% (95% CI: 14.1%, 22.1%), which was lower than the previous meta-analyses using aggregated data. Age, sex, and year of study were significantly associated with the depression z-score. Despite the additional efforts in acquiring and integrating individual data from smaller proportion of included studies, IPD meta-analysis may provide a more accurate estimation of disease burden, and allow verification of associated factors.

## Supplementary Information

**Additional file 1: Supplementary Figure 1.** PRISMA flow diagram. **Supplementary Figure 2.** Pooled prevalence of depressive symptoms computed from the reported prevalence from each included study. **Supplementary Figure 3.** Pooled prevalence of depressive symptoms for males and for females. **Supplementary Figure 4.** Pooled prevalence of depressive symptoms according to the years of study. **Supplementary Figure 5.** Pooled prevalence of depressive symptoms according to regions. **Supplementary Figure 6.** Pooled prevalence of depressive symptoms according to assessment tools.

## Data Availability

The data used to support the findings of this study are included within the article.
