# Supplementary material for: Pooled prevalence of depressive symptoms among medical students: an individual participant data meta-analysis
Source: BMC Psychiatry. 2023 Apr 14;23:251. doi: 10.1186/s12888-023-04745-5 (PMC10103433; doi:10.1186/s12888-023-04745-5)

Supplementary Figure 1 PRISMA flow diagram

10 systematic reviews / meta-analyses [Tam et al 2019]

Identified from Tam et al 2019

Supplementary Figure 2 Pooled prevalence of depressive symptoms computed from the reported prevalence from each included study

Exclusion:

Data no longer available (14)

Declined or Lost contact after first email (10)

Unable to provide the data due to loss of labelling or other reasons (14)

34 datasets from 37 studies

75 studies with responses from authors

197 studies with email addresses

249 primary studies


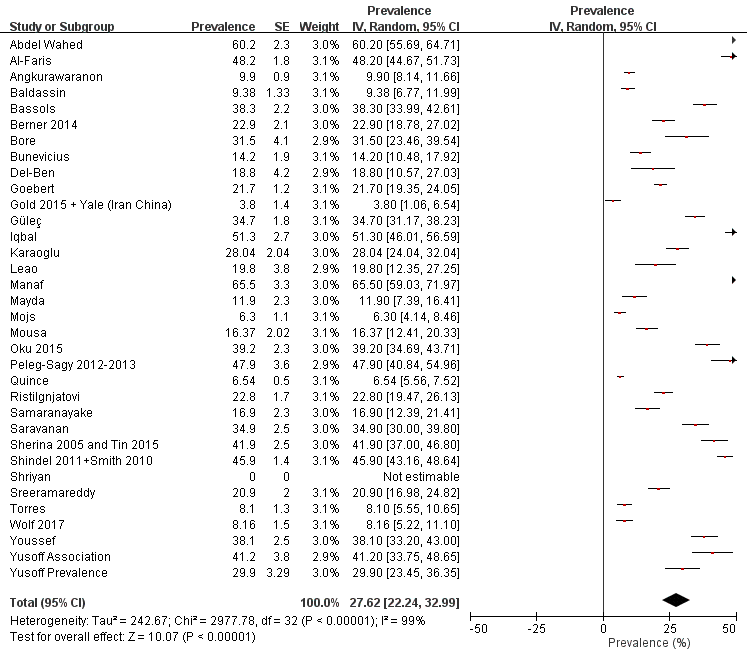


Supplementary Figure 3 Pooled prevalence of depressive symptoms for males and for females


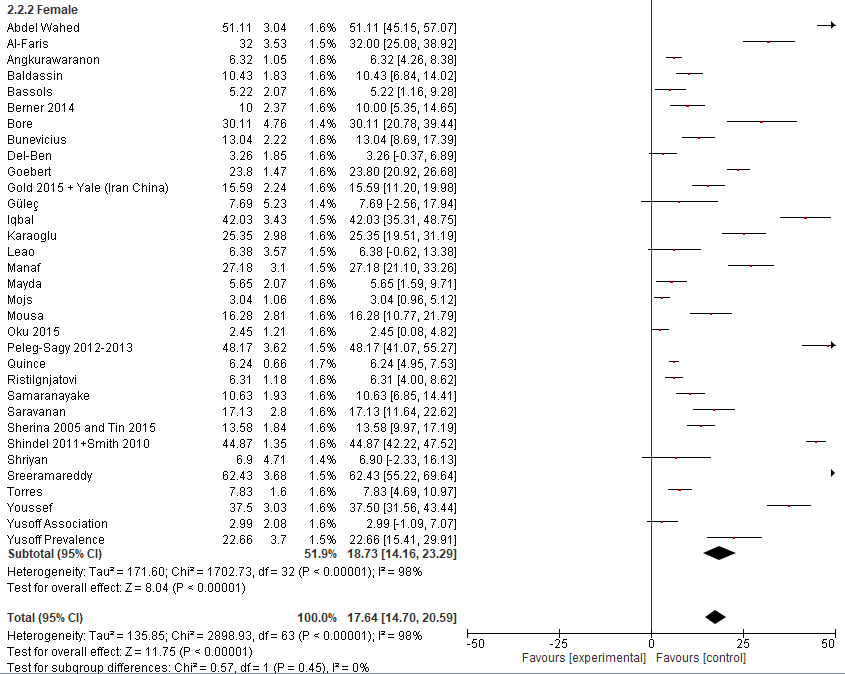

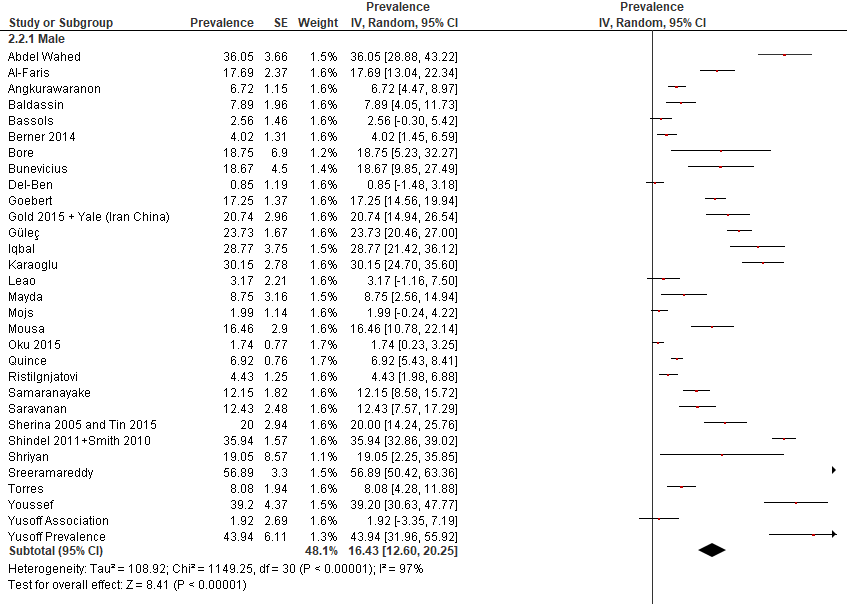


Supplementary Figure 4 Pooled prevalence of depressive symptoms according to the years of study


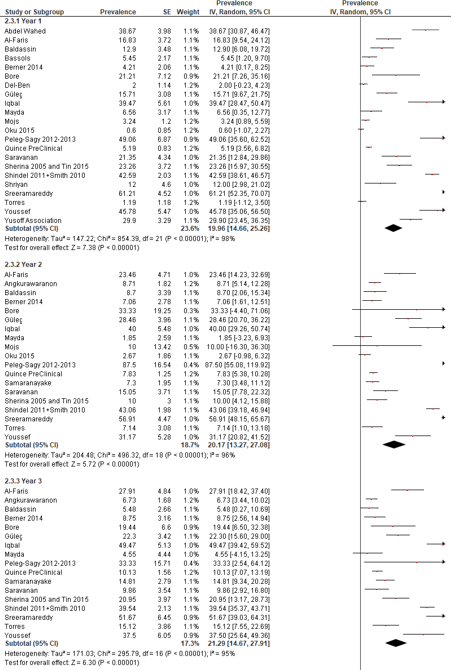

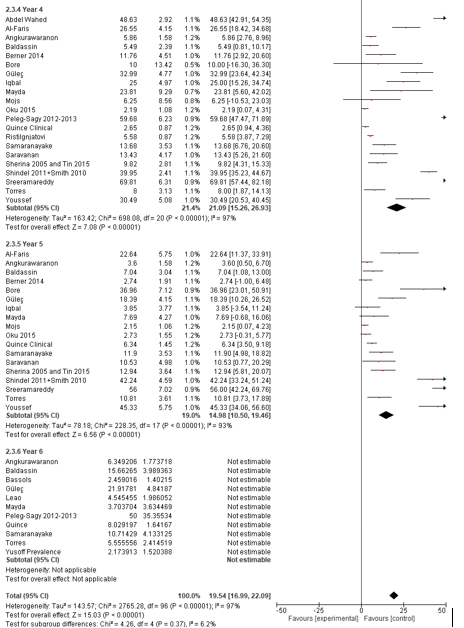


Supplementary Figure 5 Pooled prevalence of depressive symptoms according to regions


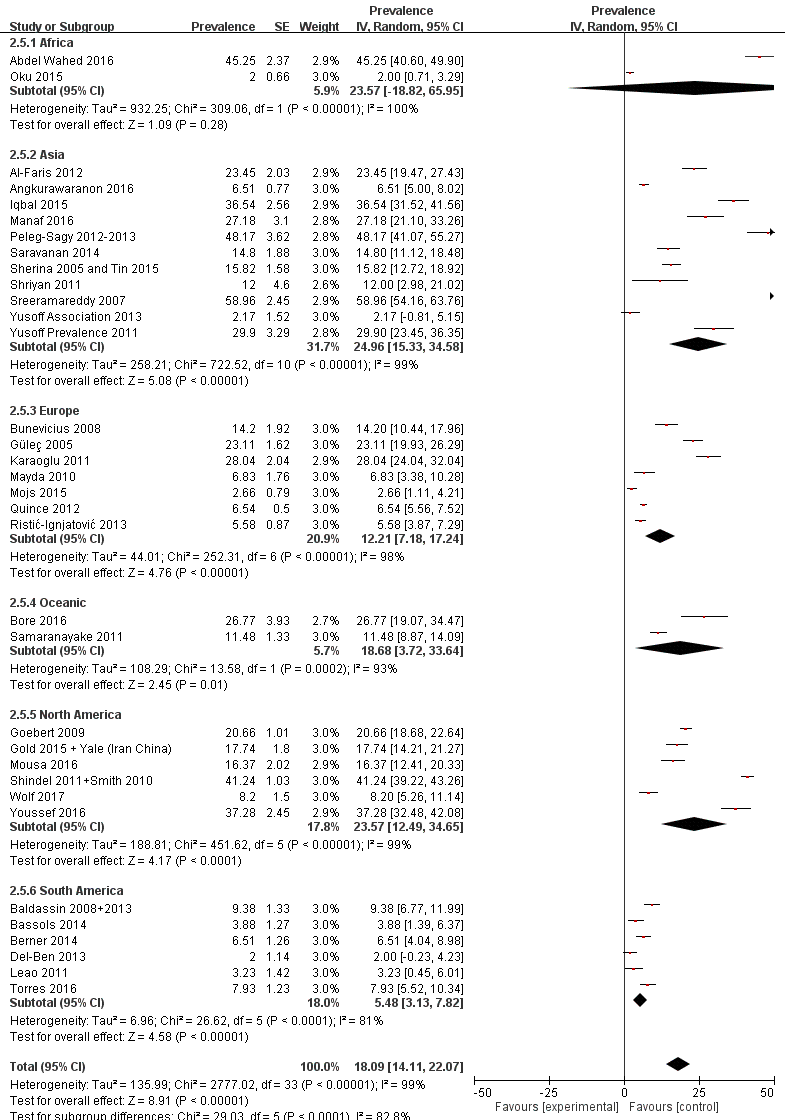


Supplementary Figure 6 Pooled prevalence of depressive symptoms according to assessment tools


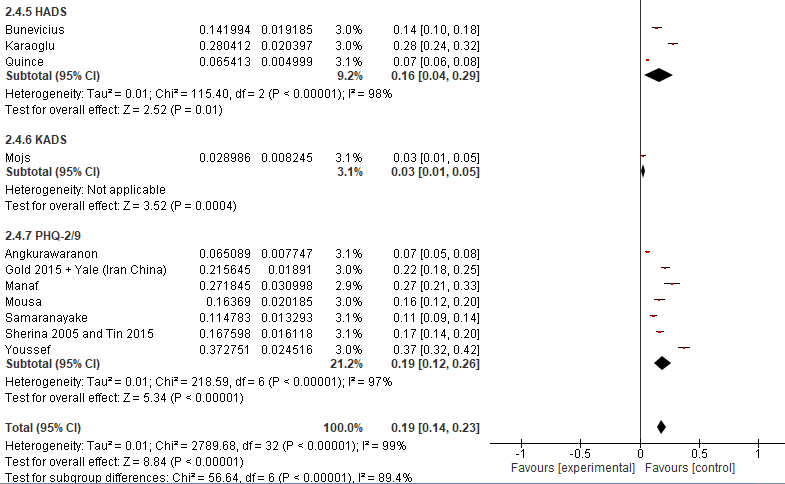

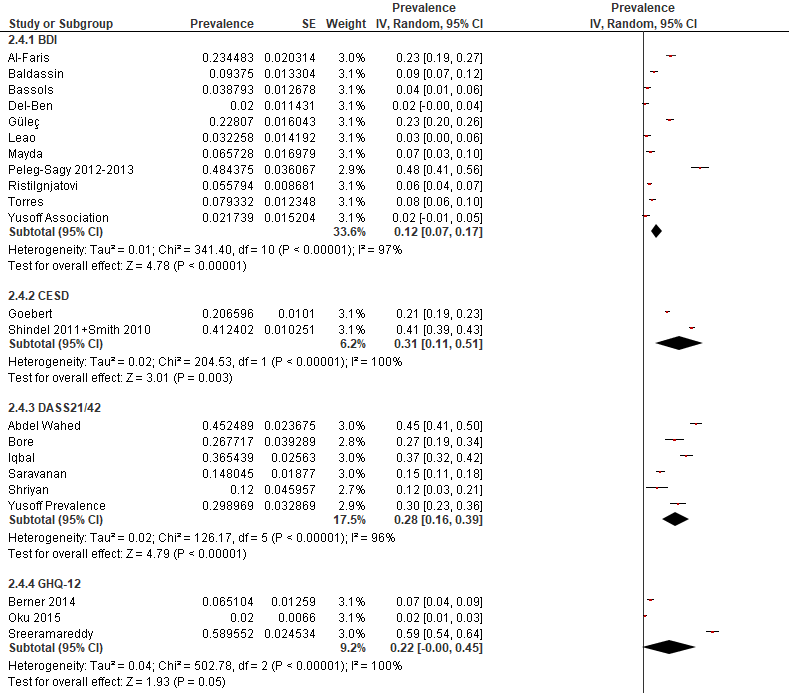

Supplement: Supplementary file 1 — Additional file 1: Supplementary Figure 1. PRISMA flow diagram. Supplementary Figure 2. Pooled prevalence of depressive symptoms computed from the reported prevalence from each included study. Supplementary Figure 3. Pooled prevalence of depressive symptoms for males and for females. Supplementary Figure 4. Pooled prevalence of depressive symptoms according to the years of study. Supplementary Figure 5. Pooled prevalence of depressive symptoms according to regions. Supplementary Figure 6. Pooled prevalence of depressive symptoms according to assessment tools. [file 12888_2023_4745_MOESM1_ESM.docx]
